# Protein Induced by Vitamin K Absence II: A Potential Biomarker to Differentiate Pancreatic Ductal Adenocarcinoma from Pancreatic Benign Lesions and Predict Vascular Invasion

**DOI:** 10.3390/jcm12082769

**Published:** 2023-04-07

**Authors:** Yang Yang, Guangbing Li, Yu Zhang, Yunfeng Cui, Jun Liu

**Affiliations:** 1 Department of Surgery, Tianjin Nankai Hospital, Nankai Clinical School of Medicine, Tianjin Medical University, Tianjin 300070, China; 2 Department of Liver Transplantation and Hepatobiliary Surgery, Shandong Provincial Hospital Affiliated to Shandong First Medical University, Jinan 250021, China

**Keywords:** protein induced by vitamin K absence II, pancreatic cancer, biomarker, diagnosis, vascular invasion

## Abstract

Background: Pancreatic ductal adenocarcinoma (PDAC) is a highly malignant gastrointestinal tumor with a poor prognosis. Serum biomarker carbohydrate antigen 19-9 (CA19-9) was the only well-established biomarker for PDAC with inadequate efficacy. This present study aimed to determine the ability of PIVKA-II to discriminate PDAC from pancreatic benign lesions and predict vascular invasion preoperatively. Methods: Patients who underwent pancreatic surgery from 2017 to 2020 were enrolled. We examined the differential diagnostic ability of protein induced by vitamin K absence II (PIVKA-II), CA19-9, and their combination and 138 with PDAC evaluated the predictive value of PIVKA-II for vascular invasion in PDAC. Methods: A total of 138 patients with PDAC and 90 patients with pancreatic benign lesions who underwent pancreatic surgery from 2017 to 2020 were enrolled. The clinicopathological characteristics were recorded. Results: There was a significant difference in levels of serum PIVKA-II between PDAC patients and patients with pancreatic benign lesions (*p* < 0.001). When the cut-off value was set to 28.9 mAU/mL according to the ROCs, the AUC, sensitivity, and specificity of PIVKA-II were 0.787, 68.1%, and 83.3%, respectively. The combined PIVKA-II and carbohydrate antigen 19-9 (CA19-9) enhanced the diagnostic accuracy, and the AUC, sensitivity, and specificity were 0.945, 87.7%, and 94.4%, respectively. PIVKA-II > 36.4 mAU/mL were independent predictive factors of vascular invasion in PDAC (*p* < 0.001). Conclusion: PIVKA-II was a potential diagnostic biomarker to differentiate PDAC from pancreatic benign lesions. PIVKA-II was complementary to CA19-9, and the combination enhanced the differential diagnostic performance. PIVKA-II > 36.4 mAU/mL was an independent predictive factor of vascular invasion in PDAC.

## 1. Introduction

Pancreatic ductal adenocarcinoma (PDAC) is a highly malignant digestive tract tumor [1]. It remains a diagnostic and therapeutic challenge as symptoms were non-specific at an early stage, and most patients are diagnosed at the non-resectable stage with multiple organ metastases or vascular invasion [2]. Consequently, the prognosis is dismal, with a 5-year survival of only 10% [1]. Accumulated evidence indicated that accurate detection enabled earlier treatment initiation, increased the possibility of surgical intervention, and improved the prognosis of patients with PDAC [3,4,5]. 

Current diagnostic modalities were based on imaging procedures and pathological examination through fine-needle aspirations, which were invasive and time-consuming [6]. Serum biomarker carbohydrate antigen 19-9 (CA19-9) was the only well-established biomarker for suspected PDAC. However, levels of CA19-9 were normal in Lewis-negative patients while elevated in patients with other tumors. It could discriminate between pancreatic cancer and healthy, with an inadequate sensitivity and specificity between 82% and 91% [7,8]. When CA19-9 was used for the distinction between cancer and benign lesions, the diagnostic efficacy was even worse: sensitivity and specificity were often no better than 65% or 60%, respectively [9]. Thus, a minimal-invasive, accurate, and real-time monitoring method for diagnosing PDAC remains a major unmet medical need.

Protein induced by Vitamin K absence II (PIVKA-II), also known as Des-gamma carboxy prothrombin (DCP), is the prothrombin precursor with the deficiency of thrombogen synthesis [10]. Clinically, PIVKA-II served as a hepatocellular carcinoma (HCC)-specific biomarker for surveillance, diagnosis, assessment of response to treatment, and prognostic prediction [11,12,13]. In recent years, it has been proposed as a promising biomarker for diagnosing patients with PDAC. Several studies suggested PIVKA-II could be used alone for pancreatic head cancer detection and had superior diagnostic performance, compared to other biomarkers [14,15]. The overexpression of PIVKA-II in pancreatic adenocarcinoma tissue was also reported [16]. Considering that the mechanism of elevated PIVKA-II and CA19-9 was independent and the levels of two biomarkers in the same patient were unrelated to each other, PIVKA-II combined with CA19-9 may offer a feasible diagnostic method for PDAC [17,18]. The promising biomarker PIVKA-II would likely not replace but complement CA19-9 with improved diagnostic performance for patients with PDAC.

Currently, radical surgical resection is the only potentially curative treatment for PDAC [19]. The assessment of vascular invasion is the most important parameter for determining resectability other than distant metastasis. However, imaging examination frequently overestimated the actual macro-vascular invasion and was limited in the prediction of micro-vascular invasion [20,21]. By contrast, PIVKA-II was reported as an angiogenic factor that was associated with the continuous growth of tumor tissues and tumor progression [13]. Studies have shown PIVKA-II could promote the secretion of other angiogenic factors and stimulate vascular endothelial cell invasion [22]. Considering its angiogenic roles, PIVKA-II might have the potential as a predictive factor for vascular invasion in PDAC and aid physicians in making clinical decisions.

In this study, we aimed to (1) assess the potential of PIVKA-II to differentiate PDAC from pancreatic benign lesions; (2) compare the diagnostic accuracy of PIVKA-II, CA19-9, and their combination; and (3) determine the ability of PIVKA-II to predict vascular invasion for PDAC.

## 2. Materials and Methods

This study was approved by the institutional ethics review board of Shandong Provincial Hospital in compliance with the Declaration of Helsinki, and the requirement for written informed consent was waived given the retrospective study design (NO. 2019–032).

### 2.1. Study Design and Population

A retrospective analysis was performed on patients with pancreatic lesions at the Department of Liver Transplantation and Hepatobiliary Surgery, Shandong Provincial Hospital, from January 2017 to December 2020. The patient information was collected from the electronic medical records. 

The inclusion criteria were as follows: (1) the first occurrence of tumor; (2) preoperatively diagnosed as pancreatic space-occupying lesions by imaging examination, postoperatively diagnosed as PDAC, or pancreatic benign diseases by pathological examination. (3) received no disease-related treatment before; (4) had complete clinical record. Patients with severe underlying diseases, Vitamin K deficiency, and hepatic diseases were excluded.

All patients underwent blood tests (e.g., blood routine, liver function, kidney function, CA19-9, and PIVKA-II) after a comprehensive history and physical examination. A contrast-enhanced abdominal CT was performed in patients with suspected pancreatic lesions. Two abdominal radiologists reviewed the CT images independently and reported the tumor location, size, and the presence of vascular invasion. Then, after the exclusion of patients with distant metastasis and severe underlying diseases, all patients underwent pancreatic surgery within 7 days. Patients with unintended distant metastasis were excluded and underwent palliative surgery. All surgical specimens were sent for pathological examination. Formalin-fixed paraffin-embedded (FFPE) tumor specimens were independently examined by two pathologists. 

Pathological staging was determined according to the 8th edition of the American Joint Committee on Cancer (AJCC) staging system [23]. Vascular invasion was defined as tumor invasion in an artery or venous vein surrounding the primary tumor and had a link between pancreatic cancer cells and vascular space through endothelial cells, consisting of macroscopic and microscopic vascular invasion (MVI). MVI was a microtubule invasion state that can only be observed around the tumor under a microscope. Patients at the stage of T4NxM0, which is referred to as PDAC with involvement of the celiac trunk, superior mesenteric artery, and/or hepatic artery, underwent palliative surgery. Metastasis only included lymph node metastasis (LNM). The standardized lymph node dissection was performed for patients undergoing radical surgery for PDAC according to the guideline of the Japanese Pancreas Society [24]. Tumor size was defined as the maximum tumor diameter. 

We retrieved clinical data, including age, sex, male, total bilirubin, type of resection, CA19-9, PIVKA-II, aspartate aminotransferase (AST), alanine aminotransferase (ALT), serum albumin, white blood cell (WBC), history of chronic pancreatitis, location of pancreatic lesions, differentiated grade, tumor size, tumor staging, and whether there were metastases, and vascular invasion.

The included patients were divided into two groups based on pathological results. We assessed the potential of PIVKA-II for differential diagnosis and compared the diagnostic accuracy of PIVKA-II, CA19-9, and their combination in patients with PDAC and pancreatic benign lesions. Then, we determined the ability of PIVKA-II to predict vascular invasion in patients with PDAC.

### 2.2. Measurements of PIVKA-II

All patients underwent blood draws according to the same standards on the day of hospitalization. Three milliliters of peripheral blood were collected into an acid citrate dextrose (ACD) tube. Then, the samples were stored at −80 °C. The content of serum PIVKA-II was measured by chemiluminescence enzyme immunoassay on an automatic chemiluminescence immunoassay instrument (LUMI-PULSE^®^ G1200, Tokyo, Japan) using Lumipulse G PIVKA-II reagent kit (FUJIREBIO Inc., Tokyo, Japan). All procedures were strictly in accordance with the instructions. 

### 2.3. Statistical Analysis

Continuous variables were expressed as median with interquartile ranges (IQR) or mean ± standard deviation (SD). Categorical variables were described as frequencies (n) with proportions (%). The chi-square test or Fisher’s exact test was performed to compare categorical variables, while the Mann–Whiney U tests were used for non-normally distributed continuous variables. Receiver operating characteristics (ROC) curves were established to determine the optimal cut-off value. The area under the curve (AUC) value was used to assess diagnostic accuracy, associated with sensitivity, specificity positive predictive value (PPV), and negative predictive value (NPV). Univariate analysis and multivariate analysis were performed using logistic regression analysis with odd ratios (OR) and estimated 95% confidence intervals (CI). IBM SPSS statistical software (version 25.0, USA) was used for all statistical analysis, and a 2-sided *p*-value < 0.05 was considered statistically significant. 

## 3. Results

### 3.1. Patients’ Characteristics

**Figure 1:**
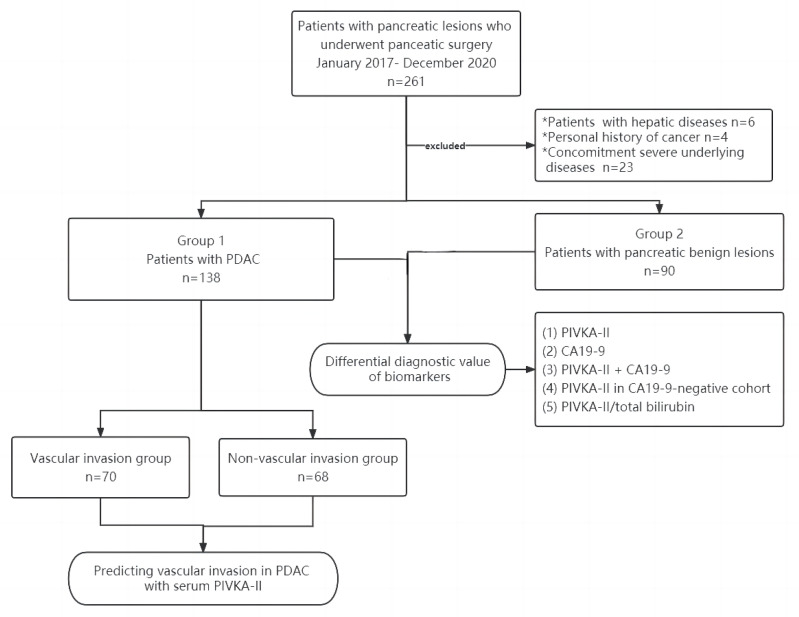
Flowchart of case selection in this study. PDAC, pancreatic ductal adenocarcinoma; CA19-9, carbohydrate antigen 19–9; PIVKA-II, protein induced by vitamin K absence-II.

We collected data from 261 patients with suspected pancreatic lesions during this study period. Based on inclusion and exclusion criteria, we excluded patients with a previous personal history of cancer (n = 4), hepatic diseases (n = 6), and severe underlying diseases (n = 23). A total of 228 patients were enrolled. The cases were divided into two groups based on pathological results. Group 1 consisted of 138 cases with pancreatic ductal adenocarcinoma. Group 2 consisting of 90 cases with pancreatic benign lesions was used as a control. In this study, pancreatic benign lesions referred to as pancreatic cystic lesions (n = 28), SPNP (n = 39), pNETs (n = 14), CP (n = 4), pancreatic hamartoma (n = 1), and IPMNs (n = 4). The flowchart of case selection in this study was presented in Figure 1.

**Figure 2:**
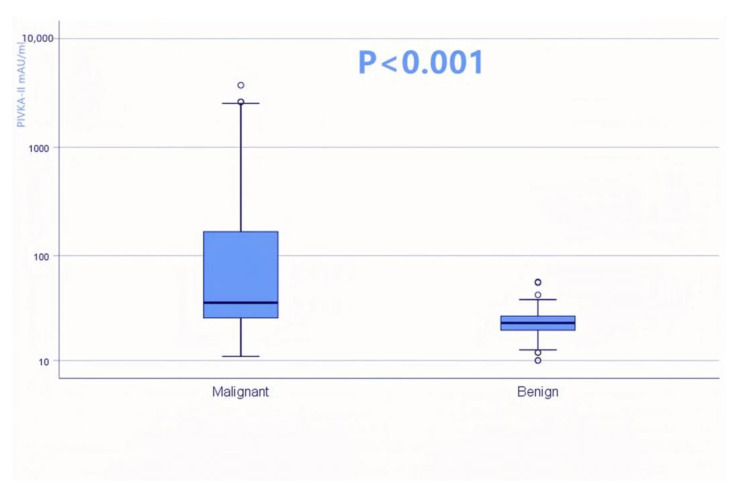
A comparison of serum protein induced by vitamin K absence-II (PIVKA-II) levels in patients with pancreatic ductal adenocarcinoma and benign lesions.

The clinical characteristics of 228 cases with pancreatic lesions were presented in Table 1. Subjects were matched by age and sex (*p* = 0.352; *p* = 0.457, respectively). A total of 31 patients (13.6%) had a previous history of pancreatitis. Group1 had higher levels of AST (median 39.5 IU/L versus 20 IU/L, *p* < 0.001), ALT (median 55 IU/L versus 16 IU/L, *p* < 0.001), and total bilirubin (median 23.6 mg/dL versus 12.5 mg/dL, *p* < 0.001) than Group 2, while Group 2 had a higher level of serum albumin (3.6 ± 0.1 g/dL versus 3.9 ± 0.1 g/dL, *p* = 0.027). The serum levels of PIVKA-II in patients with PDAC and pancreatic benign lesions were significantly different (Figure 2). The serum levels of CA19-9 and PIVKA-II are significantly higher in PDAC patients (median 379.3 U/L versus 10.6 U/L, *p* < 0.001; median 36.4 mAU/mL versus 23.3 mAU/mL, *p* < 0.001, respectively). 

The histopathological features were presented in Table 2. In PDAC patients, 36 (26.1%) patients had a poorly differentiated grade, 57 (41.3%) patients had lymphatic metastasis, 84 (60.9%) patients had tumor size more than 4cm, and 7969 (50.70%) patients were in the presence of vascular invasion, consisting of 44 (31.9%) patients with macro-vascular invasion and 26 (18.8%) patients with micro-vascular invasion. A total of 10 (7.2%) patients were at the stage of T4NxM0, which referred to pancreatic ductal adenocarcinoma without distant metastases, with involvement of the celiac trunk, superior mesenteric artery, and/or hepatic artery. Additionally, 54 (39.1%) patients with PDAC underwent vascular resection and or reconstruction based on the combination of the imaging findings and corresponding morphological features obtained during the operation, and 92 (66.7%) patients had negative surgical margins. In the control cohort, 33 (36.67%) with tumors located in the pancreatic head and 42 (46.67%) patients had tumors more than 4 cm. 

### 3.2. Differential Diagnostic Value of PIVKA-II in PDAC

**Figure 3:**
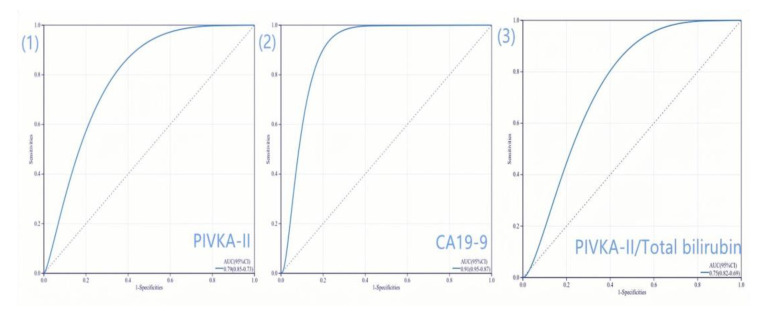
ROC curves for pancreatic ductal adenocarcinoma diagnosis. (**1**) ROC curve of PIVKA-II for PDAC diagnosis; (**2**) ROC curve of CA19-9 for PDAC diagnosis; (**3**) ROC curve of PIVKA-II/Total bilirubin for PDAC diagnosis. ROC, Receiver operating characteristics; PIVKA-II, protein induced by vitamin K absence-II, CA19-9, carbohydrate antigen 19-9; AUC, the area under the curve; PDAC, pancreatic ductal adenocarcinoma.

The diagnostic value of PIVKA-II to differentiate PDAC from pancreatic benign lesions was assessed using the ROC curve [Figure 3(1)]. The optimal cut-off value of PIVKA-II was determined as 28.9 mAU/mL using the Youden index. The AUC of PIVKA-II was 0.789 (95% CI, 0.730–0.845). At the cut-off value, the sensitivity, specificity, PPV, and NPV of PIVKA-II were 68.1%, 83.3%, 86.2%, and 63.0%, respectively. 

### 3.3. Comparison of Differential Diagnostic Value of PIVKA-II, CA19-9, and Their Combination in PDAC

**Figure 4:**
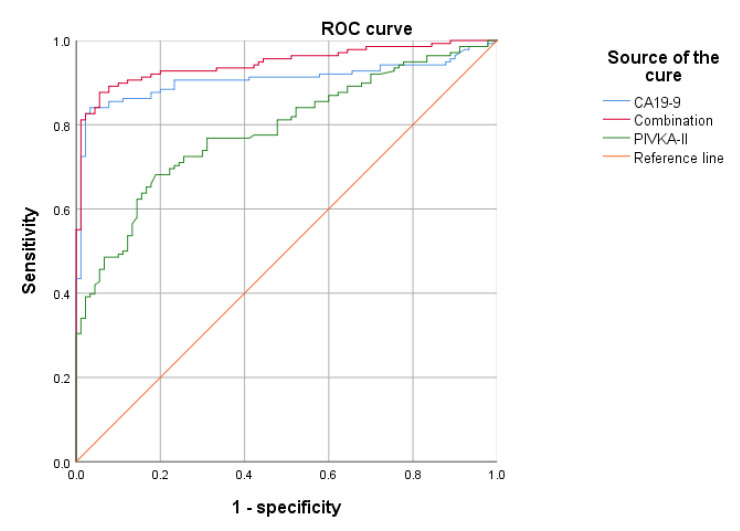
ROC curves of CA19-9, PIVKA-II, and their combination for distinguishing pancreatic ductal adenocarcinoma. ROC, Receiver operating characteristics; PIVKA-II, protein induced by vitamin K absence-II; CA19-9, carbohydrate antigen 19–9.

According to Figure 3(2), when the optimal cut-off value of CA19-9 was 39.5 U/L, the AUC, sensitivity, specificity, PPV, and NPV for PDAC differential diagnosis were 0.906 (0.864–0.948), 83.3%, 94.4%, 95.8%, and 78.7%, respectively. Compared to PIVKA-II alone, CA19-9 had better differential diagnostic efficacy. Then, we compared the diagnostic accuracy of PIVKA-II, CA19-9, and their combination in PDAC using ROC curves (Figure 4). Combined PIVKA-II and CA19-9 had superior diagnostic efficacy for patients with PDAC. The AUC and sensitivity were improved to 87.7% and 0.945 (95% CI, 0.916–0.974), respectively, with specificity unchanged. The results were presented in Table 3.

### 3.4. Differential Diagnostic Value of PIVKA-II in CA19-9-Negative Cohort

We evaluated the differential diagnostic value of PIVKA-II as a complementary tool to the measurement of CA19-9 with CA19-9 < 39.5 U/L. The study included 23 patients with PDAC and 85 patients with pancreatic benign lesions. When the cut-off value of PIVKA-II was set as 28.9 mAU/mL, the sensitivity and specificity of the PDAC differential diagnosis were 65.2% and 83.5%, respectively (Table 4). The difference in the diagnostic performance of PIVKA-II between the normal population and the CA19-9-negative population was within 5%, which was considered acceptable.

### 3.5. Differential Diagnostic Value of Adjusted PIVKA-II in PDAC

To exclude the potential interference of jaundice, we attempted to adjust the preoperative serum PIVKA-II value by dividing it by the total bilirubin levels and compared the adjusted PIVKA-II with PIVKA-II alone for their diagnostic value in patients with PDAC. The AUC of adjusted PIVKA-II was 0.749 (95% CI, 0.686–0.813), and the sensitivity and specificity of adjusted PIVKA-II for PDAC diagnosis was 66.7% and 78.9%, respectively, using the ROC curve [Figure 3(3)]. The difference in diagnostic performance between the adjusted PIVKA-II and PIVKA-II alone was within 5%, which was considered acceptable (Table 3).

### 3.6. Predictive Value of PIVKA-II for Vascular Invasion in PDAC

**Figure 5:**
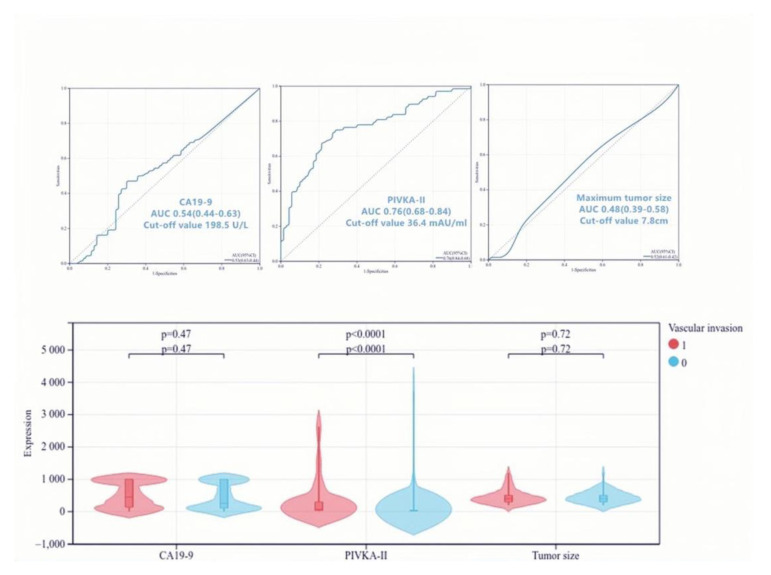
Cut-off values of PIVKA-II, CA19-9, and tumor size for predicting vascular invasion in pancreatic ductal adenocarcinoma. CA19-9 levels had no significant difference between patients with and without vascular invasion (*p* = 0.47). The ROCs showed the cut-off value of CA19-9 was 198.5 U/L, and the AUC was 0.54 (95% CI, 0.44–0.63). PIVKA-II levels significantly differed between patients with and without vascular invasion (*p* < 0.0001). The ROCs showed the cut-off value of PIVKA-II was 36.4 mAU/mL, and the AUC was 0.76 (95% CI, 0.68–0.84). Tumor size had no significant difference between patients with and without vascular invasion (*p* = 0.72). The ROCs showed the cut-off value of maximum tumor size was 7.8 cm, and the AUC was 0.48 (95% CI, 0.39–0.58). AUC is the area under the curve; ROC is receiver operating characteristics; CI is confidence interval; PIVKA-II is protein induced by vitamin K absence-II; CA19-9 is carbohydrate antigen 19-9.

**Figure 6:**
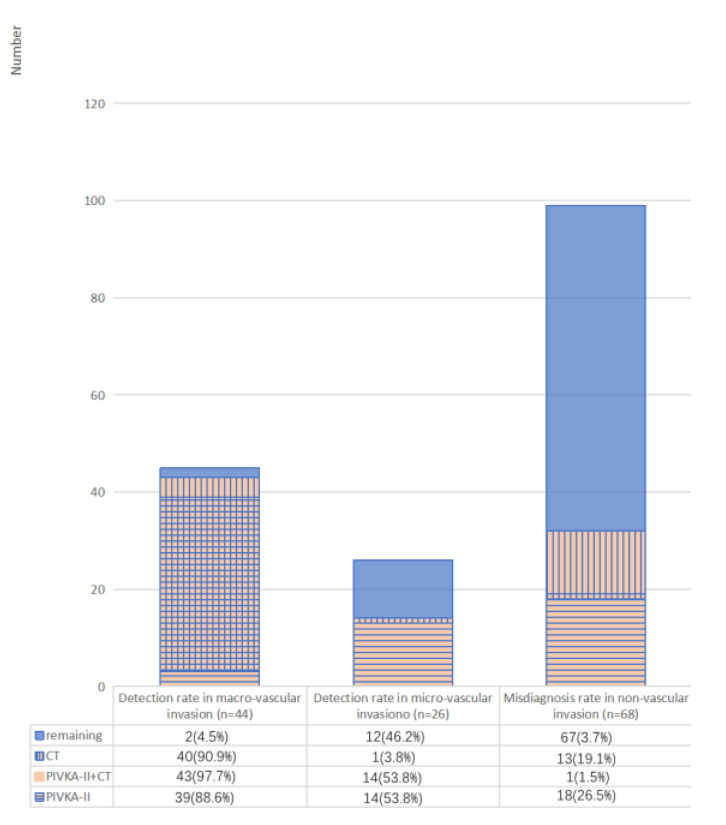
Diagnostic efficacy of PIVKA-II, CT examination and their combination for vascular invasion in PDAC. When we evaluate the diagnostic efficacy of combined CT examination and PIVKA-II for vascular invasion in PDAC, single positive was defined as positive and double negative was defined as negative. PIVKA-II, Protein induced by vitamin K absence-II; CT, computed tomography; PDAC, pancreatic ductal adenocarcinoma.

A total of 70 cases were identified with vascular invasion by pathological examinations, consisting of 44 cases with macro-vascular invasion and 26 cases with micro-vascular invasion. The optimal cut-off values of PIVKA-II, CA19-9, and tumor size were 36.4 mAU/mL, 198.5 U/L, and 7.8 cm using ROCs (Figure 5). The results of the univariate and multivariate analysis were listed in Table 5. Univariate analysis demonstrated that PIVKA-II > 36.4 mAU/mL (*p* < 0.001), CA19-9 > 198.5 U/L (*p* = 0.039), tumor size (*p* = 0.032), and preoperative imaging findings (*p* < 0.001) were significant predictors of vascular invasion in PDAC. PIVKA-II > 36.4 mAU/mL (OR = 0.07; 95% CI, 0.03–0.21; *p* < 0.001) and preoperative imaging findings (OR = 0.09; 95% CI, 0.03–0.26; *p* < 0.001) were independent predictors of vascular invasion in PDAC by multivariate analysis. According to Figure 6, compared to PIVKA-II, CT examination provided a higher sensitivity for the detection of macro-vascular invasion (88.6% versus 90.9%) and a lower misdiagnosis rate of PDAC (19.1% versus 26.5%). However, in terms of micro-vascular invasion, PIVKA-II had better diagnostic performance (sensitivity, 53.8% versus 3.8%). Then, we examined the effect of the combined CT examination and PIVKA-II, where a single positive was defined as positive and a double negative was defined as negative. Compared to using CT examination alone, the detection rates of combined PIVKA-II with CT examination for the macro- and micro-vascular invasion were improved to 97.7% and 53.8%, respectively, and the misdiagnosis rate of PDAC was reduced to 1.5%.

## 4. Discussion

We evaluated serum PIVKA-II as a potential biomarker to differentiate pancreatic ductal adenocarcinoma from pancreatic benign lesions and identify vascular invasion. The results demonstrated that using PIVKA-II alone was inadequate for PDAC differential diagnosis. PIVKA-II and CA19-9 were complementary biomarkers, and PIVKA-II combined with CA19-9 could improve the differential diagnostic accuracy for PDAC. Of note, preoperative PIVKA-II > 36.4 mAU/mL was an independent factor associated with vascular invasion in PDAC.

Currently, differential diagnosis of PDAC and pancreatic benign lesions is based on imaging techniques, fine-needle biopsy, and serum biomarkers [4]. Imaging techniques including abdominal ultrasound (US), computed tomography (CT), magnetic resonance imaging (MRI), and endoscopic ultrasonography are operator dependent, technology dependent, and time consuming [25]. Biopsy tissues, which are considered the gold standard, were limited for clinical examination with a high risk of bleeding and tumor cell dissemination. Given the advantages of being non-invasive, objective, and reproducible, using serum biomarkers are an attractive tool for the management of PDAC. Biomarkers induced by the changes in genetic, transcriptomic, metabolomic, and proteomic levels were proposed for PDAC differential diagnosis [25,26,27]. 

CA19-9 is the only well-established serum biomarker for PDAC diagnosis and prognosis. However, 5–10% of patients with pancreatic cancer were Lewis antigen-negative, resulting in the inability to synthesize CA19-9 [28,29,30]. In addition, in terms of the false positive results existing in cases of pancreatitis, liver cirrhosis, cholangitis, and other digestive cancers, the utility of CA19-9 in clinical practice is limited, with a reported sensitivity and specificity between 82% and 91% [7,8]. When CA19-9 was used for the distinction between cancer and benign lesions, the diagnostic efficacy was even worse: sensitivity and specificity were often no better than 65% or 60%, respectively [9,31]. In recent years, many potential diagnostic biomarkers have been investigated, but none of them were superior to CA19-9 [32,33]. Thus, a minimal-invasive, accurate, and real-time monitoring method for differential diagnosis of PDAC and pancreatic benign lesions remains a major unmet medical need.

PIVKA-II, known as Des-gamma carboxyprothrombin (DCP), was considered a biomarker for surveillance, detection, prognostic prediction, and treatment assessment in HCC [12,13]. Recent studies suggested serum and tissue levels of PIVKA-II were both elevated in PDAC [14,16]. Hepatoid differentiation of tumors has been speculated as one of the mechanisms of PIVKA-II production in PDAC [34]. Based on that, we attempted to investigate the differential diagnostic value of PIVKA-II in 138 patients with PDAC and 90 patients with pancreatic benign lesions. At the optimal cut-off value of 28.9 mAU/mL, the sensitivity and specificity of PIVKA-II were 68.1% and 83.3%, respectively. The results indicated that the diagnostic accuracy of using PIVKA-II alone was inadequate. Our results were inconsistent with the previous studies. In 2019, Tartaglione, S. et al. reported that the sensitivity and specificity of PIVKA-II for PDAC were 78.67% and 90.67%, respectively, when the cut-off value was set as 48 mAU/mL according to their internal measurements [15]. However, the sample size was small with a total of 46 patients. The discrepancy might be due to ethnic differences and the size of the study population. Our study included a larger number of patients and controls with a total of 228 patients and determined the optimal cut-off value using ROC curves.

In 2018, a biomarker signature (9 metabolites and additionally CA19-9) was identified for the differential diagnosis between PDAC and chronic pancreatitis with a sensitivity of 89.9% (95% CI 81.0–95.5%) and a specificity of 91.3% (95% CI 82.8–96.4%) [35]. It showed the feasibility of biomarkers combined with CA19-9 to distinguish malignant pancreatic lesions. Then, biomarkers complementing CA19.9 were evaluated. Considering that the mechanism of PIVKA-II and CA19-9 was independent and the levels of two biomarkers in the same patient were unrelated to each other, PIVKA-II complementary to CA19-9 might improve the diagnostic performance in PDAC [17,18]. A previous study reported that PIVKA-II had better performance compared to CA19-9, carcinoembryonic antigen (CEA), and CA242 for PDAC detection [15]. There are no reports on the combined PIVKA-II and CA19-9. In this present study, we evaluated the differential diagnostic value of PIVKA-II, CA19-9, and their combination. The results showed the combined PIVKA-II and CA19-9 enhanced the diagnostic performance in the differential diagnosis of PDAC and pancreatic benign lesions (sensitivity, 87.7%; specificity, 94.4%; AUC, 0.945; 95% CI, 0.916–0.974). Furthermore, we examined the efficacy of PIVKA-II in the CA19-9-negative cohort to verify the conclusion. The results were consistent with the conclusion. This confirmed that PIVKA-II and CA19-9 are complementary to distinguish PDAC from pancreatic benign lesions.

Evidence indicated that serum levels of PIVKA-II were elevated in many conditions, including Vitamin K deficiency, malnutrition, drug, acute hepatic failure, alcoholic liver disease, and other tumors [17]. Thus, we excluded patients with Vitamin K deficiency, other liver diseases, and other tumors. In addition, studies indicated jaundice could disturb the levels of PIVKA-II [8]. The pancreatic head tumor may present obstructive jaundice due to its location, causing false-positive results. Excluding the interference of jaundice remains an issue to address. Kemik, A.S. et al. evaluated the diagnostic accuracy of PIVKA-II in patients with pancreatic head adenocarcinoma. They suggested serum PIVKA-II could be used alone [14]. In this current study, we attempted to adjust the preoperative serum PIVKA-II value by dividing it by the total bilirubin levels and compared the adjusted PIVKA-II with PIVKA-II alone for their diagnostic value in patients with PDAC. The difference in diagnostic performance between the adjusted PIVKA-II and PIVKA-II alone was within 5%, which was considered acceptable. 

PDAC is associated with a poor prognosis, with a 5-year survival of only 5% [3]. Radical surgical resection is the only potentially curative treatment for PDAC [19]. However, due to the lack of effective early diagnostic tools for PDAC, most patients are diagnosed at the non-resectable stage with a 15–20% R0 resection rate [36]. 

Severe vascular invasion makes radical surgery impossible, thus, the assessment of vascular invasion is the most important parameter for determining resectability other than distant metastasis and avoiding unnecessary surgery in those with the unresectable disease for pancreatic cancer [37]. 

Imaging examination is currently the primary means of assessing efficacy. It is known that abdominal and pelvic multidetector computed tomography (CT) is optimal for the pancreatic radiological investigation to assess local macrovascular invasion [20]. A frequent error is misdiagnosing an involved major vessel. According to NCCN Guidelines, the PPV of CT were 80.8%, which means 20% of patients with pancreatic cancer missed the radical surgical opportunity [20]. On the other hand, it is not possible to differentiate microvascular invasion using the radiological modality. As a result, about 60% of resected cases with arterial resections had histologic arterial infiltration [38,39]. The previous studies that reported neoadjuvant chemotherapy appear to have a potential benefit in borderline resectable pancreatic cancer with microvascular invasion [38,39]. In order to not deny the opportunity for radical surgery in patients with borderline resectable disease, accurate preoperative assessment of vascular invasion in addition to imaging modality in PDAC is particularly needed [40].

Studies reported elevated PIVKA-II was associated with tumor progression and angiogenesis through the KDR-PLC-γ-MAPK signaling pathway [41,42]. PIVKA-II could promote the secretion of other angiogenic factors in tumor cells and stimulate invasion in vascular endothelial cells [22,43]. In this current study, we evaluated PIVKA-II as a predictor for accurate preoperative assessment of vascular invasion and found that PIVKA-II > 36.4 mAU/mL was an independent predictor of vascular invasion in PDAC. The combined PIVKA-II and imaging findings could provide comprehensive information for determining vascular invasion and selecting the optimal strategy. Compared to using CT examination alone, the detection rates of combined PIVKA-II with CT examination for macro- and micro-vascular invasion were improved to 97.7% and 53.8%, respectively, and the misdiagnosis rate of PDAC was reduced to 1.5%.

In addition, studies reported CA19-9 could assess stage, prognosis, resectability, tumor recurrence, and therapeutic efficacy [44,45,46]. However, in our study, CA19-9 was only significant in univariate analyses but not in multivariate analyses, which may be due to the expanded definition of vascular invasion consisting of macro- and micro-vascular invasion. 

This present study has several limitations. The first is selection bias in the retrospective study. Second, it was a single-centered study, and large-scale, prospective, and multicenter studies are needed. Third, although we attempted to exclude the interference of jaundice by dividing the PIVKA-II value by the bilirubin level in patients with jaundice, further study in patients without jaundice is needed to confirm our findings. 

## 5. Conclusions

PIVKA-II is a potential diagnostic biomarker to differentiate PDAC from pancreatic benign lesions. The combined PIVKA-II and CA19-9 enhanced the differential diagnostic performance. PIVKA-II > 36.4 mAU/mL is an independent predictive factor of vascular invasion in PDAC.

## Figures and Tables

**Table 1:** Clinical characteristics of the patients.

	Characteristic	Group 1 n = 138		Group 2 n = 90	*p*
Age, years, median (IQR)		62 (52–67)		60 (50–66)	*p* = 0.352
Sex, male, n (%)		91 (65.9%)		55 (60.0%)	*p* = 0.457
Total bilirubin, mg/dL, median (IQR)		23.6 (13.3–185.4)		12.5 (9.3–15.9)	*p* < 0.001
Type of resection PD n (%)		100 (72.5%)		33 (36.7%)	*p* < 0.001
CA19-9 U/L median (IQR)		379.3 (114.1–1000)		10.6 (6.8–15.7)	*p* < 0.001
PIVKA-II mAU/mL, median (IQR)		36.4 (26.1–166.8)		23.3 (19.9–27.2)	*p* < 0.001
AST IU/L median (IQR)		39.5 (21–111.5)		20 (16.3–24)	*p* < 0.001
ALT IU/L median (IQR)		55 (17–185.8)		16 (11.3–23)	*p* < 0.001
Albumin (g/dL) mean ± SD		3.6 ± 0.1	3.9 ± 0.1	*p* = 0.027	
WBC × 10^9^/L median (IQR)		5.64 (4.8–7.2)		5.7 (4.9–6.5)	*p* = 0.696
	CP n (%)	14 (10.1%)		17 (18.9%)	*p* = 0.060

IQR, interquartile range; PD, pancreaticoduodenectomy; CA19-9, carbohydrate antigen 19-9; PIVKA-II, protein induced by vitamin K absence-II; AST, aspartate aminotransferase; ALT, alanine aminotransferase; WBC, white blood cell; CP, chronic pancreatitis; SD, standard deviation.

**Table 2:** Histopathological features of pancreatic lesions.

Variables	Group 1 (n%)	Group 2 (n%)
Location (pancreatic head)	100 (72.5%)	33 (36.67%)
Differentiated grade (poor),	36 (26.1%)	NA
Lymphatic metastasis	57 (41.3%)	NA
Vascular invasion	7069 (50.70%)	NA
Macro-	44 (31.9%)	NA
Micro-	26 (18.8%)	NA
Vascular resection and or reconstruction	54 (39.1%)	NA
Tumor size *, >4 cm	84 (60.9%)	42 (46.67%)
Staging, T4NxM0 ^+^	10 (7.2%)	NA
Vascular resection and or reconstruction	54 (39.1%)	NA
R0 resection ^#^	92 (66.7%)	NA

* Tumor size was defined as the maximum tumor diameter. ^+^ T4NxM0 referred to pancreatic ductal adenocarcinoma without distant metastases, with involvement of the celiac trunk, superior mesenteric artery, and/or hepatic artery. ^#^ R0 resection was defined as resection with tumor-free margins on pathological assessment. NA, not applicable.

**Table 3:** Sensitivity, specificity, PPV, and NPV of different biomarkers in discriminating PDAC from pancreatic benign lesions.

Variables	Sensitivity	Specificity	AUC (95%CI)	PPV	NPV
PIVKA-II	68.1%	83.3%	0.787 (0.730–0.845)	86.2%	63.0%
CA19-9	83.3%	94.4%	0.906 (0.864–0.948)	95.8%	78.7%
PIVKA-II + CA19-9	87.7%	94.4%	0.945 (0.916–0.974)	96.0%	83.3%
PIVKA-II/ Total bilirubin	66.7%	79.2%	0.749 (0.686–0.813)	82.9%	60.7%

AUC, Area under the curve; CI, Confidence interval; PPV, Positive predictive value; NPV, Negative predictive value; PIVKA-II, Protein induced by vitamin K absence-II; CA19-9, carbohydrate antigen 19-9; PDAC, pancreatic ductal adenocarcinoma.

**Table 4:** The differential diagnostic value of PIVKA-II in CA19-9-negative patients.

	Group 1	Group 2	Total	*p*	Sensitivity	Specificity
PIVKA-II(+)	15	14	29	<0.001	65.2%	83.5%
PIVKA-II(−)	8	71	79			
Total	23	85	108			

PIVKA-II, protein induced by vitamin K absence-II; *p*, *p*-value.

**Table 5:** Univariate analysis and multivariate analysis for predictors of vascular invasion in pancreatic ductal adenocarcinoma.

Characteristic	Vascular Invasion		Univariate Analysis		Multivariate Analysis	
	Positive n = 70	Negative n = 68	*p* Value	OR (95% CI)	*p* Value	OR (95% CI)
PIVKA-II, >I36.4 mAU/mL, n	51	18	<0.001	7.46 (3.51–15.84)	<0.001	0.07 (0.03–0.21)
Age, ≥70 years, n	8	12	0.300	0.60 (0.23–2.58)	NA	NA
Sex, male, n	47	44	0.763	1.12 (0.56–2.25)	NA	NA
CA19-9. >198.5 U/L, n	49	36	0.039	2.07 (1.03–4.17)	0.73	0.85 (0.35–2.10)
Albumin <3.2 g/dl, n	22	30	0.124	0.58 (0.29–1.16)	NA	NA
Tumor size * >7.8 cm, n	9	2	0.032	4.87 (1.01–23.43)	0.10	0.19 (0.03–1.39)
Location, pancreatic head, n	54	46	0.212	1.61 (0.76–3.43)	NA	NA
Pathologic differentiation, poorly, n	20	16	0.500	1.30 (0.61–2.79)	NA	NA
Imaging findings, positive, n	41	13	<0.001	5.98 (2.77–12.91)	<0.001	0.09 (0.03–0.26)

* Tumor size was defined as the maximum tumor diameter. PIVKA-II, protein induced by vitamin K absence-II; CA19-9 carbohydrate antigen; OR, odds ratio; CI, confidence interval; NA, not applicable.

## Data Availability

The datasets analyzed during this current study are available from the corresponding author upon reasonable request.
